# A phase-field model for vesicle membranes incorporating area-difference elasticity

**DOI:** 10.1371/journal.pcbi.1014185

**Published:** 2026-04-17

**Authors:** Yihong Liang, Emine Celiker, Ping Lin

**Affiliations:** 1 School of Mathematics and Physics, University of Sciences and Technology Beijing, Beijing, China; 2 School of Engineering, University of Leicester, Leicester, United Kingdom; 3 Division of Mathematics, University of Dundee, Dundee, United Kingdom; George Washington University, UNITED STATES OF AMERICA

## Abstract

This paper presents a phase-field model for simulating the three-dimensional deformation of vesicle membranes, incorporating area-difference elasticity (i.e., the elasticity arising from the difference between the inner and outer lipid leaflets), with constraints on bulk volume and surface area. We develop efficient numerical schemes based on the Fourier-spectral method for spatial discretization and temporal evolution. The model successfully captures a wide variety of steady-state vesicle shapes. The numerical experiments demonstrate that by tuning the simulation parameters, the vesicle can transition from a simple spherical and discocyte shape to complete membrane fission, asymmetric pear shaped structures, as well as complex multi-armed starfish-like and nested configuration. These results highlight the crucial role of area-difference elasticity in determining vesicle morphology.

## Author summary

Biological membranes act as the protective envelopes of cells, and their ability to dynamically change shape is crucial for vital functions like transporting nutrients, cell division, and engulfing pathogens. To understand these complex behaviours, scientists often study vesicles — simplified, artificial membrane sacs. Predicting how vesicles deform in three dimensions is notoriously difficult due to the microscopic complexity of their bilayer structures.

While previous studies have highlighted the importance of “area-difference elasticity”(ADE), earlier predictions of vesicle morphology typically utilized sharp-interface models. A major limitation of the sharp-interface approach is that it requires complex parametrisation, making it mathematically difficult to handle and simulate spontaneous topological changes, such as membrane budding or vesiculation.

While phase-field models, which use a smooth function to represent the membrane interface, can naturally handle dynamic topological changes without explicit interface tracking, incorporating the ADE constraint introduces severe mathematical stiffness. In this study, we developed a robust and highly efficient computational framework to overcome these numerical challenges.

By employing this mathematical approach, our model successfully reproduces a wide spectrum of realistic vesicle morphologies, ranging from simple discocyte shape to topological membrane division, as well as highly complex, multi-armed “starfish” and nested configurations. Crucially, to the best of our knowledge, this robust framework captures the asymmetric pear shape for the first time within a phase-field model — a classic morphology previously only predicted using sharp-interface models. Ultimately, our work not only provides deeper theoretical insights into the physical rules governing cellular membranes but also offers a powerful computational tool for guiding future applications in targeted drug delivery and the design of biomimetic materials.

## Introduction

Biological membranes, which define the boundaries of cells and most internal organelles, are crucial for isolating cellular internal structures from the external environment. They have long been a focus of considerable interest among biologists, chemists, and physicists [1–3]. Typically composed of phospholipids and proteins, these membranes form a highly organized bilayer structure. At the molecular level, this structure displays extraordinary complexity and exhibits various dynamic properties. As a consequence of this complex structure, the mathematical modelling and simulation of the dynamic shape changes can be very challenging. Hence, the construction of efficient mathematical formulations and numerical methods is still a necessity.

To better understand the physicochemical properties of biological membranes and their physiological functions, vesicles are often used as model systems [4,5]. Studying vesicles not only deepens our understanding of biological membranes but also facilitates advances in biomimetic research [6]. The processes of vesicle formation and evolution, including complex phenomena like budding and vesiculation, are intimately linked with their physicochemical properties. Experimental studies on the mechanisms underlying vesicle formation have been explored in references [7–9].

Over the past few decades, numerous theoretical models have been proposed to describe the bending behavior of biological membranes. A groundbreaking contribution by Canham, Evans and Helfrich [10–12] introduced the classic Helfrich bending energy model, also known as the sharp-interface elastic bending energy. This model describes the total energy of the membrane in terms of the square of its mean curvature and a Gaussian curvature term. It has been instrumental in explaining the shapes of biological structures, such as red blood cells. The general elastic bending energy is derived from the Hooke’s Law:


$$E=\int_{\Gamma }\left(a_{1}+a_{2}(H-c_{0}{)}^{2}+a_{3}G\right)ds,$$ (1)

where *a*_1_ represents the surface tension, which accounts for the interaction effects between the vesicle material and its surrounding fluid, $H=\frac{c_{1}+c_{2}}{2}$ is the mean curvature of the membrane surface, with *c*_1_ and *c*_2_ as the principal curvatures, and *G* = *c*_1_*c*_2_ is the Gaussian curvature. The parameters *a*_2_ and *a*_3_ represent the bending rigidities, determined by the properties of the materials forming the membrane. The spontaneous curvature is denoted as *c*_0_. This model and its variants have been widely applied to study various phenomena, including vesicle deformation and protein insertion into membranes. Recently, advanced data-driven computational approaches, such as neural-network-based solvers [13], have also been developed to efficiently predict vesicle equilibrium shapes governed by the Helfrich model. However, challenges arise in simulating topological changes, such as budding or fusion, as the sharp-interface approach requires complex parameterizations that are difficult to handle numerically [14,15].

To overcome these challenges, Du and his collaborators proposed a phase-field-based mathematical model to simulate membrane deformation driven by bending energy [16–20]. They used a simplified form of the bending energy of the form


$$E_{elastic}=\frac{\kappa }{2}\int_{\Gamma }(H-c_{0}{)}^{2}ds.$$ (2)

The phase-field model uses a smooth phase field function to naturally represent the membrane interface, avoiding the difficulties associated with explicit interface tracking and facilitating spontaneous topological changes [16–21]. However, their model did not account for the area difference between the inner and outer leaflets of the membrane.

Among the various membrane properties, area-difference elasticity plays a crucial role in determining the shape and stability of vesicles [22]. The elastic behavior of a bilayer vesicle is governed by both its bending rigidity and constraints on the membrane’s surface area and volume. The ADE model [4,22] suggests that the area difference between the inner and outer leaflets of the lipid bilayer is a key determinant of the vesicle’s shape. The energy functional for this model is defined as:


$$E_{ADE}=E_{elastic}+\frac{\overset{―}{\kappa }}{2}\frac{\pi }{AD^{2}}(\Delta A-\Delta A_{0}{)}^{2}.$$ (3)

Here, $\Delta A_{0}$ is the relaxed area difference between the two leaflets of the plasma membrane and $\bar{\kappa }$ is a constant representing the bending elastic moduli of the vesicle. The parameter *A* represents the total membrane area, which is assumed to be constant based on the initial condition for the vesicle. The area difference ΔA arises from membrane curvature or variations in molecular composition, imposing energetic constraints on the vesicle’s shape and dynamics [23]. Thus, research into vesicles incorporating ADE not only reveals the physical mechanisms governing vesicle-related biological processes but also provides theoretical guidance for applications such as drug delivery, biomimetic material design, and biomechanics [5,6,23–25].

While the application of phase-field models to the Helfrich bending energy is a well-established and highly successful field [16–21], the existing literature predominantly focuses on models restricted by simple global constraints, such as bulk volume and surface area. These standard constraints are mathematically characterized by lower-order local integrals. In contrast, incorporating the Area-Difference Elasticity (ADE) model presents a fundamental mathematical challenge. The ADE functional requires the global integration of the mean curvature. The square of this global integral introduces severe mathematical stiffness and strongly coupled non-local non-linearities in its variational derivative.

To address this challenge, this work systematically incorporates the non-local ADE functional into the phase-field modeling of vesicle membranes. Specifically, building upon existing theoretical frameworks, we derive a consistent phase-field formulation for the ADE energy and verify the existence of its minimizers. More importantly, to overcome the severe mathematical stiffness introduced by the highly non-linear ADE variational derivative, we develop an efficient numerical framework optimized for 3D simulations. Ultimately, we demonstrate that this model effectively captures a wide spectrum of complex morphological and topological transitions driven by the delicate interplay between bending energy and area-difference constraints. Notably, through extensive parameter exploration, our robust framework successfully reproduces the asymmetric pear shape — a classic morphology previously predicted by Miao et al. [4,22] using sharp-interface models — for the first time within a phase-field approach.

The remainder of this paper is organized as follows. In the section *Methods*, we detail the phase-field formulation incorporating the ADE term, outline the tailored numerical schemes, and provide the theoretical justification for the energy minimizers. The section *Results* is devoted to numerical experiments, demonstrating the model’s capability to capture various complex morphological and topological transformations. We further interpret the physical significance of these findings in the section *Discussion*. Finally, concluding remarks are given in the *Conclusion*.

## Methods

First, we introduce a phase function ϕ(x), defined on the computational domain Ω used to label the inside and outside of the vesicle Γ. The level set {x:ϕ(x)=0} represents the membrane, while {x:ϕ(x)>0} represents the interior of the membrane and {x:ϕ(x)<0} the exterior. We define the following modified elastic energy:


$$W(\phi )=\int_{\Omega }\frac{\kappa \epsilon }{2}{\left|\Delta \phi -\frac{1}{\epsilon^{2}}(\phi^{2}-1)(\phi +C\epsilon )\right|}^{2}dx,$$ (4)

where $\Omega \in {𝐑}^{3}$, ϵ is defined as the vesicle membrane thickness and *C* is $\sqrt{2}$ times the spontaneous curvature *c*_0_. κ is the known bending elastic moduli [20].

Following [20], the energy (4) asymptotically converges to the Helfrich-type bending energy (2), up to a constant factor. The detailed derivation can be found in [20] and is omitted here for brevity.

Moreover, the following functional


$$V(\phi )=\int_{\Omega }\frac{\phi (x)+1}{2}dx$$ (5)

goes to the volume. Given the functional


$$B(\phi )=\int_{\Omega }\frac{\epsilon }{2}|\nabla \phi {|}^{2}+\frac{1}{4\epsilon }(\phi^{2}-1{)}^{2}dx,$$ (6)

the surface area of Γ can be denoted as $A(\phi )=\frac{3\sqrt{2}}{4}B(\phi )$.

Next, the phase-field formulation of the second term in (3) is required, namely


$$\frac{\overset{―}{\kappa }}{2}\frac{\pi }{AD^{2}}(\Delta A-\Delta A_{0}{)}^{2}.$$ (7)

As defined in *Introduction*, $\bar{\kappa }$ is a constant representing the bending elastic moduli of the vesicle. The parameter *A* represents the total membrane area, which is conserved during the shape evolution, and $\Delta A_{0}$, defined as the relaxed area difference between the inner and outer leaflets of the plasma membrane, serves as a crucial parameter.

In [23], the two leaflets of a closed bilayer with fixed interleaflet separation *D* are required by geometry to differ in area by an amount


$$\Delta A=D\int d𝒜(c_{1}+c_{2})=D\int d𝒜2H.$$ (8)

The parameter *D* holds a clear physical meaning: it is the separation distance between the neutral surfaces of the two leaflets composing the bilayer, corresponding to roughly two-thirds the total bilayer thickness [22], *c*_1_ and *c*_2_ are the two local principal curvatures, and $H=\frac{c1+c2}{2}$ is the mean curvature. In the phase-field formulation, the integral for ΔA, originally defined on the membrane surface in (8), is extended to the entire computational domain Ω:


$$\Delta A=2D\int_{\Omega }Hd\Omega .$$

Consequently, a phase-field representation of the mean curvature *H* is required.

Based on the proof given in [18], since


$$H=-\frac{\sqrt{2}\epsilon }{2(1-\phi^{2})}\left(\Delta \phi +\frac{1}{\epsilon^{2}}\phi (1-\phi^{2})\right),$$

and


$$\int_{-\infty }^{+\infty }(1-\phi^{2}{)}^{2}dx=\frac{4\sqrt{2}\epsilon }{3},$$

we have


$$\begin{array}{cc} \Delta A & \sim 2D\times \frac{3}{4\sqrt{2}\epsilon }\int_{\Omega }(1-\phi^{2}{)}^{2}\times \frac{-\epsilon }{\sqrt{2}(1-\phi^{2})}\left(\Delta \phi +\frac{1}{\epsilon^{2}}\phi (1-\phi^{2})\right)dx\\ & =-\frac{3D}{4}\int_{\Omega }\left((1-\phi^{2})\Delta \phi +\frac{1}{\epsilon^{2}}\phi (1-\phi^{2}{)}^{2}\right)dx \end{array}$$ (9)

Hence, the second term of equation (3) is redefined as


$$\begin{array}{cc} G(\phi ): & =\frac{\overset{―}{\kappa }}{2}\frac{\pi }{A_{0}D^{2}}(\Delta A-\Delta A_{0}{)}^{2}\\ & =\frac{\overset{―}{\kappa }}{2}\frac{\pi }{A_{0}D^{2}}{\left(-\frac{3D}{4}\int_{\Omega }\left((1-\phi^{2})\Delta \phi +\frac{1}{\epsilon^{2}}\phi (1-\phi^{2}{)}^{2}\right)dx-\Delta A_{0}\right)}^{2}, \end{array}$$ (10)

and


$$E_{ADE}(\phi )=W(\phi )+G(\phi ).$$

The following Proposition 1 is adapted from Proposition 3.1 in [21], where they derived the existence of a minimizer for the phase-field model with elastic bending energy. We have extended this result to incorporate the ADE term in our model, which is essential for capturing the behavior of vesicle membranes in our formulation.

**Proposition 1.** *Let S denote the feasible set of* $\phi \in H^{2}(\Omega )$ *such that* V(ϕ)=α *and* A(ϕ)=β*. If for some suitable* α *and* β*, S is non-empty, then there exists a* $\phi^{*}\in S$ *minimizing* $E_{ADE}(\phi )$*.*

*Proof.* The energy functional is always non-negative and thus is bounded from below, and there exists a minimizing sequence $\{\phi_{n}\in S{\}}_{n=1}^{\infty }$, such that


$$\lim_{n\to \infty }E_{ADE}(\phi )=C^{*},$$

where C^*^ is the infimum of *E*_*ADE*_.

From $B(\phi_{n})=\beta$, we derive


$$\int_{\Omega }\frac{\epsilon }{2}|\nabla \phi {|}^{2}dx\leq \beta ,\;\;\;\int_{\Omega }\frac{1}{4\epsilon }(\phi^{2}-1{)}^{2}dx\leq \beta ,$$

therefore


$$\|\nabla \phi_{n}{\|}_{L^{2}}^{2}\leq \frac{2\beta }{\epsilon },\;\;\;\|\phi_{n}^{2}-1{\|}_{L^{2}}^{2}\leq 4\epsilon \beta .$$

Let ${\bar{\phi }}_{n}=\frac{\int_{\Omega }\phi_{n}dx}{\left|\Omega \right|}=\frac{2\alpha }{\left|\Omega \right|}-1$. Using $V(\phi_{n})=\alpha$ and Poincaré-Wirtinger inequality, we have


$$\|\phi_{n}-{\bar{\phi }}_{n}{\|}_{L^{2}}\leq C_{p}\|\nabla \phi_{n}{\|}_{L^{2}}\leq C_{p}\sqrt{\frac{2\beta }{\epsilon }},$$

where *C*_*p*_ is the Poincaré constant. Consequently,


$$\|\phi_{n}{\|}_{L^{2}}\leq \|\phi_{n}-{\bar{\phi }}_{n}{\|}_{L^{2}}+\|{\bar{\phi }}_{n}{\|}_{L^{2}}\leq C_{p}\sqrt{\frac{2\beta }{\epsilon }}+\left(\frac{2\alpha }{\left|\Omega \right|}-1\right)\sqrt{\left|\Omega \right|}.$$

Thus $\phi_{n}$ is uniformly bounded in $H^{1}(\Omega )$, that is


$$\|\phi_{n}{\|}_{H^{1}}\leq C_{1},\forall n,\text{where}C_{1}dependson\}\;\alpha ,\;\beta ,\;\epsilon \text{ and }|\Omega |.$$

Define $q(\phi )=\frac{1}{\epsilon^{2}}(\phi^{2}-1)(\phi +C\epsilon )$. Since $W(\phi_{n})\leq E_{ADE}(\phi_{n})\to C^{*}$, we have


$$\|\Delta \phi_{n}-q(\phi_{n}){\|}_{L^{2}}^{2}\leq C_{2},\forall n.$$

Considering the Sobolev embedding theorem,


$$\|\phi_{n}{\|}_{L^{6}}\leq C_{s}\|\phi_{n}{\|}_{H^{1}}\leq C_{s}C_{1}.$$

we have


$$|q(\phi_{n})|\leq \frac{1}{\epsilon^{2}}|\phi_{n}^{2}-1||\phi_{n}|+\frac{C}{\epsilon }|\phi_{n}^{2}-1|.$$

By Hölder’s inequality, we obtain


$$\|(\phi_{n}^{2}-1)\phi_{n}{\|}_{L^{2}}\leq \|\phi_{n}^{2}-1{\|}_{L^{3}}\|\phi_{n}{\|}_{L^{6}}.$$

From $\|\phi_{n}^{2}{\|}_{L^{3}}=\|\phi_{n}{\|}_{L^{6}}\leq C_{s}C_{1}$, we get


$$\|\phi_{n}^{2}-1{\|}_{L^{3}}\leq (C_{s}C_{1}{)}^{2}+|\Omega {|}^{\frac{1}{3}}.$$

So $\|(\phi_{n}^{2}-1)\phi_{n}{\|}_{L^{2}}\leq \left((C_{s}C_{1}{)}^{2}+|\Omega {|}^{\frac{1}{3}}\right)C_{s}C_{1}≜C_{3}^{\prime }$. Then


$$\|q(\phi_{n}){\|}_{L^{2}}\leq \frac{C_{3}^{\prime }}{\epsilon^{2}}+2C\sqrt{\frac{\beta }{\epsilon }}≜C_{3}.$$

Therefore, $\Delta \phi_{n}$ is uniformly bounded in $L^{2}(\Omega )$, namely


$$\|\Delta \phi_{n}{\|}_{L^{2}}\leq \|\Delta \phi_{n}-q(\phi_{n}){\|}_{L^{2}}+\|q(\phi_{n}){\|}_{L^{2}}\leq C_{2}+C_{3}≜C_{4}.$$

From the *H*^2^-regularity theory for elliptic problems, we have $\phi_{n}$ uniformly bounded in $H^{2}(\Omega )$, i.e., $\|\phi_{n}{\|}_{H^{2}}\leq C_{6},\forall n$.

By uniform boundedness in $H^{2}(\Omega )$, there exists a subsequence still denoted $\phi_{n}$ and $\phi \in H^{2}(\Omega )$ such that $\phi_{n}⇀\phi^{*}$ in $H^{2}(\Omega )$. The Rellich-Kondrachov compact embedding theorem implies $\phi_{n}\to \phi^{*}$ in $H^{1}(\Omega )$. Furthermore, since $H^{1}(\Omega )↪L^{p}(\Omega )$ and $H^{2}(\Omega )↪C^{0,m}(\Omega )(m>0)$, we have $\phi_{n}\to \phi^{*}$ in $L^{p}(\Omega )(p<\infty )$ and uniformly in $C(\bar{\Omega })$. Strong convergence in $L^{1}(\Omega )$ gives


$$V(\phi^{*})=\int_{\Omega }\phi^{*}dx=\lim_{n\to \infty }\int_{\Omega }\phi_{n}dx=\alpha .$$

Strong convergence in $H^{1}(\Omega )$ and uniform convergence yield:


$$A(\phi^{*})=\lim_{n\to \infty }A(\phi_{n})=\beta .$$

Thus $\phi^{*}\in S$.

Weak convergence in $H^{2}(\Omega )$ implies $\Delta \phi_{n}⇀\Delta \phi^{*}$ in $L^{2}(\Omega )$. Uniform convergence $\phi_{n}\to \phi^{*}$ in $C(\bar{\Omega })$ gives $q(\phi_{n})\to q(\phi^{*})$ in *L*^∞^. Thus


$$\Delta \phi_{n}-q(\phi_{n})⇀\Delta \phi^{*}-q(\phi^{*})\;\;in\;\;L^{2}(\Omega ).$$

By weak lower semicontinuity of the *L*^2^-norm, we have


$$\|\Delta \phi^{*}-q(\phi^{*}){\|}_{L^{2}}^{2}\leq {lim inf}_{n\to \infty }\|\Delta \phi_{n}-q(\phi_{n}){\|}_{L^{2}}^{2}.$$

Therefore


$$W(\phi^{*})\leq {lim inf}_{n\to \infty }W(\phi_{n}).$$

Define the functional


$$J(\phi )=-\frac{3D}{4}\int_{\Omega }\left((1-\phi^{2})\Delta \phi +\frac{1}{\epsilon^{2}}\phi (1-\phi^{2}{)}^{2}\right)dx-\Delta A_{0},$$

so that $G(\phi )=\frac{\overset{―}{\kappa }}{2}\frac{\pi }{A_{0}D^{2}}J(\phi {)}^{2}$. From uniform convergence $\phi_{n}\to \phi^{*}$ in $C(\bar{\Omega })$ and weak convergence $\Delta \phi_{n}⇀\Delta \phi^{*}$ in $L^{2}(\Omega )$, we have


$$1-\phi_{n}^{2}\to 1-(\phi^{*}{)}^{2},\;\;\phi_{n}(1-\phi_{n}^{2}{)}^{2}\to \phi^{*}{\left(1-(\phi^{*}{)}^{2}\right)}^{2}\;\;\text{in}\;\;L^{\infty }(\Omega ).$$

According to the property of strong-weak convergence of products, it implies $J(\phi_{n})\to J(\phi^{*})$, and consequently $G(\phi^{*})\to G(\phi^{*})$. Combining these results, we obtain


$$\begin{array}{cc} E_{ADE}(\phi^{*}) & =W(\phi^{*})+G(\phi^{*})\\ & \leq {lim inf}_{n\to \infty }W(\phi_{n})+\lim_{n\to \infty }G(\phi_{n})\\ & ={lim inf}_{n\to \infty }\left(W(\phi_{n})+G(\phi_{n})\right)\\ & ={lim inf}_{n\to \infty }E(\phi_{n})=C^{*}. \end{array}$$

This shows that $\phi^{*}\in S$ is a minimizer of *E*_*ADE*_ and satisfies the constraints. □

In order to numerically enforce these two constraints, we introduce two corresponding penalty terms in the free energy so that the total energy is


$$E_{M}(\phi )=W(\phi )+G(\phi )+M_{1}{\left(V(\phi )-\alpha \right)}^{2}+M_{2}{\left(A(\phi )-\beta \right)}^{2},$$ (11)

where *M*_1_, *M*_2_ are the penalty coefficients, α is the fixed initial volume of Ω, β is the surface area constraint, G(ϕ) essentially acts as a global energetic penalty term to enforce the preferred area difference constraint, functionally similar to the volume and surface area constraints.

For simplicity of notation, let us take *M*_1_ = *M*_2_ = *M*, we then have the following existence theorem:

**Theorem 1.** *For any M > 0, there exists* $\phi_{M}\in H^{2}(\Omega )$ *such that*


$$E_{M}(\phi_{M})=\inf_{\phi \in H^{2}\Omega }E_{M}(\phi ).$$

The proof is similar to the one in Proposition 1, so we omit the details. To make it more closely tied to the proposed formulation, we now prove Theorem 2, which presents a result based on the framework of Du and Wang [22]. The conclusions in [22] can be generalized to the current model incorporating area-difference elasticity (ADE).

**Theorem 2.** *With S non-empty, the minimizer* $\phi^{*}$ *of* $E_{ADE}(\phi )$ *in S can be approximated by the minimizer* $\phi_{M}$ *of* $E_{M}(\phi )$*, that is, there exists a sequence* $\phi_{M_{n}}$*, which are minimizers of* $E_{M_{n}}$*, converging to some minimizer* $\phi^{*}$ *of* $E_{ADE}(\phi )$ *in* $H^{2}(\Omega )$ *and satisfying*


$$E_{ADE}(\phi^{*})=\lim_{M_{n}\to \infty }E_{M_{n}}(\phi_{M_{n}}).$$

*Proof.* ∀M>0,


$$E_{M}(\phi_{M})=\min E_{M}(\phi )\leq E_{M}(\phi^{*})=E_{ADE}(\phi^{*}).$$

Thus, $V(\phi_{M})$, $A(\phi_{M})$ and $E_{ADE}(\phi_{M})$ are uniformly bounded for large *M*. Similar to the proof of Proposition 1, there exists a subsequence of $\phi_{M_{n}}$, such that $\phi_{M_{n}}⇀\tilde{\phi }$ in $H^{2}(\Omega )$ and $\phi_{M}\to \tilde{\phi }$ in $H^{1}(\Omega )$. Then


$$V\left(\tilde{\phi }\right)=\lim_{n\to \infty }V(\phi_{M_{n}})=\alpha ,$$

$$A\left(\tilde{\phi }\right)=\lim_{n\to \infty }A(\phi_{M_{n}})=\beta ,$$

Thus $\tilde{\phi }\in S$. By convergence and semi-lower continuity, we have


$$E_{ADE}\left(\tilde{\phi }\right)\leq \lim_{n\to \infty }E_{ADE}(\phi_{M_{n}})\leq \lim_{n\to \infty }E_{M_{n}}(\phi_{M_{n}})\leq E_{ADE}(\phi^{*})=\min_{\phi \in S}E_{ADE}(\phi ).$$

So $\tilde{\phi }$ achieves the minimum of $E_{ADE}(\phi )$ with volume and surface area constraints. Moreover, the inequality becomes an equality, which shows that $\phi_{M_{n}}$ strongly converges to $\tilde{\phi }$.□

Therefore, we see that the original problem of minimizing the energy of the ADE model with prescribed surface area and bulk volume constraints can be formulated as finding the function ϕ=ϕ(x) on the whole domain that minimizes the energy E(ϕ).

Let


$$T_{1}(\phi )=M_{1}{\left(V(\phi )-\alpha \right)}^{2},\;T_{2}(\phi )=M_{2}{\left(A(\phi )-\beta \right)}^{2},$$

The Allen-Cahn type dynamic equation takes the following form:


$$\phi_{t}=-\frac{\delta E_{M}}{\delta \phi }=-\left(\frac{\delta W}{\delta \phi }+\frac{\delta G}{\delta \phi }+\frac{\delta T1}{\delta \phi }+\frac{\delta T2}{\delta \phi }\right).$$ (12)

with the initial value $\phi (x,0)=\phi_{0}(x)$. For the boundary conditions, we adopt periodic boundary conditions. While other approaches, such as a Dirichlet condition (ϕ=−1) is used in [16], can effectively confine a vesicle, our choice is motivated by two key factors. Computationally, periodic boundary conditions are a natural requirement for the highly efficient Fourier-spectral method employed in our numerical schemes. Physically, this condition is well-justified for modeling a representative vesicle in a bulk environment, thereby avoiding artificial interactions with solid domain walls, especially when the vesicle occupies a significant portion of the domain.

Let us denote


$$\begin{array}{c} \left\{ \begin{array}{cc} & f=\epsilon \Delta \phi -\frac{1}{\epsilon }(\phi^{2}-1)\phi ,\\ & f_{c}=\epsilon \Delta \phi -\frac{1}{\epsilon }(\phi^{2}-1)(\phi +C\epsilon ),\\ & g=\Delta f_{c}-\frac{1}{\epsilon^{2}}(3\phi^{2}+2C\epsilon \phi -1)f_{c}. \end{array}\right. \end{array}$$

We provide the required variational derivatives:


$$\begin{array}{cc} & \frac{\delta W}{\delta \phi }=\kappa g,\\ & \frac{\delta G}{\delta \phi }=-\frac{3\overset{―}{\kappa }\pi }{4A_{0}D}(\Delta A-\Delta A_{0})\left(-2\phi \Delta \phi -\Delta (\phi^{2})+\frac{1}{\epsilon^{2}}(1-6\phi^{2}+5\phi^{4})\right):=h,\\ & \frac{\delta T_{1}}{\delta \phi }=M_{1}\left(V(\phi )-\alpha \right),\\ & \frac{\delta T_{2}}{\delta \phi }=\frac{3\sqrt{2}}{2}M_{2}\left(A(\phi )-\beta \right)(-f). \end{array}$$

Next, we focus on the numerical solution of (12). For the spatial discretization, we use Fourier spectral methods. Due to the regularization effect of the finite transition layer, for fixed ϵ, the solutions exhibit high-order regularities. This property makes spectral methods, often implemented with FFT routines, very efficient for this problem. There are a number of options for the time discretization. One can use the forward Euler method:


$$\frac{\phi_{n+1}-\phi_{n}}{\Delta t}=-\left(\kappa g(\phi_{n})+\frac{\delta G(\phi_{n})}{\delta \phi }+\frac{\delta T1(\phi_{n})}{\delta \phi }+\frac{\delta T2(\phi_{n})}{\delta \phi }\right).$$ (13)

The energy decay properties can be ensured, but only for a sufficiently small time step Δt. To improve the stability and accuracy of the temporal approximations while maintaining comparable efficiency, we can apply a semi-implicit time discretization scheme:


$$\frac{\phi_{n+1}-\phi_{n}}{\Delta t}=-\left(\kappa g_{n,n+1}+\frac{\delta G(\phi_{n})}{\delta \phi }+\frac{\delta T1(\phi_{n})}{\delta \phi }+\frac{\delta T2(\phi_{n})}{\delta \phi }\right),$$ (14)

where


$$\begin{array}{cc} g_{n,n+1}= & \epsilon \Delta^{2}\phi_{n+1}+\frac{2}{\epsilon }\Delta \phi_{n+1}-\frac{1}{\epsilon }\Delta \phi_{n}^{3}-C\Delta \phi_{n}^{2}-\frac{3}{\epsilon }\phi_{n}^{2}\Delta \phi_{n}-2C\phi_{n}\Delta \phi_{n}\\ & +\frac{1}{\epsilon^{3}}(3\phi_{n}^{2}+2C\epsilon \phi_{n}-1)(\phi_{n}^{2}-1)(\phi_{n}+C\epsilon ). \end{array}$$

In this scheme, the higher-order derivative terms in ϕ are treated implicitly, while the remaining nonlinear parts are treated explicitly.

Theoretically, we can also adopt a fully implicit scheme


$$\begin{array}{cc} \frac{\phi_{n+1}-\phi_{n}}{\Delta t}= & -\{\kappa g(\phi_{n},\phi_{n+1})+h(\phi_{n},\phi_{n+1})+\frac{M_{1}}{2}\left(V(\phi_{n+1})+V(\phi_{n})-2\alpha \right)\\ & +\frac{3\sqrt{2}M_{2}}{4}\left(A(\phi_{n+1})+A(\phi_{n})-2\beta \right)\left(-f(\phi_{n},\phi_{n+1})\right)\} \end{array}$$ (15)

and ensure the monotonic decreasing of the energy while preserving the constraints. First, we define the function *f*, *g* and *h* as


$$\begin{array}{cc} & f(\phi ,\eta )=\frac{\epsilon }{2}\Delta (\phi +\eta )-\frac{1}{4\epsilon }(\phi^{2}+\eta^{2}-2)(\phi +\eta ),\\ & g(\phi ,\eta )=\frac{1}{2}\Delta \left(f_{c}(\phi )+f_{c}(\eta )\right)-\frac{1}{2\epsilon^{2}}\left(\phi^{2}+\phi \eta +\eta^{2}+C\epsilon (\phi +\eta )-1\right)\left(f_{c}(\phi )+f_{c}(\eta )\right),\\ & h(\phi ,\eta )=-\frac{3\overset{―}{\kappa }\pi }{8A_{0}D}\left(\Delta A(\phi_{n})+\Delta A(\phi_{n+1})-2\Delta A_{0}\right)\\ & \;\;\;\;\times \left(-\phi \Delta \phi -\eta \Delta \eta -\frac{1}{2}\Delta (\phi^{2}+\eta^{2})+\frac{1}{\epsilon^{2}}(1-2\phi^{2}-2\eta^{2}-2\phi \eta +2\phi^{4}+2\eta^{4}+\phi^{2}\eta^{2})\right). \end{array}$$

These reformulated functions exhibit symmetry in both arguments. This generalization of nonlinear terms in frameworks provides convenience for our analysis.

**Lemma 1.** *The solution of the fully implicit scheme* (15) *satisfies the discrete energy law:*


$$E_{M}(\phi_{n+1})-E_{M}(\phi_{n})+\frac{1}{\Delta t}\int_{\Omega }(\phi_{n+1}-\phi_{n}{)}^{2}dx=0.$$

*Proof.* For (15), we have


$$W(\phi_{n+1})-W(\phi_{n})=\int_{\Omega }(\phi_{n+1}-\phi_{n})\kappa g(\phi_{n+1},\phi_{n})dx,$$ (16)

$$G(\phi_{n+1})-G(\phi_{n})=\int_{\Omega }(\phi_{n+1}-\phi_{n})h(\phi_{n+1},\phi_{n})dx,$$ (17)

$$\begin{array}{cc} & M_{1}\left(V(\phi_{n+1}\right)-\alpha {)}^{2}-M_{1}{\left(V(\phi_{n})-\alpha \right)}^{2}\\ = & M_{1}\left(V(\phi_{n+1})+V(\phi_{n})-2\alpha \right)\left(V(\phi_{n+1})-V(\phi_{n})\right)\\ = & M_{1}\left(V(\phi_{n+1})+V(\phi_{n})-2\alpha \right)\int_{\Omega }(\phi_{n+1}-\phi_{n})\frac{1}{2}dx, \end{array}$$ (18)

$$\begin{array}{cc} & M_{2}{\left(A(\phi_{n+1})-\beta \right)}^{2}-M_{2}{\left(A(\phi_{n})-\beta \right)}^{2}\\ = & M_{2}\left(A(\phi_{n+1})+A(\phi_{n})-2\beta \right)\left(A(\phi_{n+1})-A(\phi_{n})\right)\\ = & M_{2}\left(A(\phi_{n+1})+A(\phi_{n})-2\beta \right)\int_{\Omega }(\phi_{n+1}-\phi_{n})\left(-\frac{3\sqrt{2}}{4}f(\phi_{n+1},\phi_{n})\right)dx. \end{array}$$ (19)

Putting together (16)-(19), we have the discrete energy law for the numerical solution corresponding to (15). □

**Remark 1.** *While Lemma 1 establishes the unconditional stability for the fully implicit scheme (15), we employ the semi-implicit scheme (14) in our numerical experiments for its superior computational efficiency. Although a rigorous theoretical proof for (14) is omitted, its energy stability is verified numerically, as shown in the Results section “Numerical Accuracy and Energy Stability”.*

We may also adopt a full backward Euler scheme


$$\frac{\phi_{n+1}-\phi_{n}}{\Delta t}=-\left(\kappa g(\phi_{n+1})+\frac{\delta G(\phi_{n+1})}{\delta \phi }+\frac{\delta T1(\phi_{n+1})}{\delta \phi }+\frac{\delta T2(\phi_{n+1})}{\delta \phi }\right).$$ (20)

As a result, the discrete energy law no longer holds in its strict form, and instead, we obtain the following.

**Proposition 2.** *For all* Δt>0 *and a given* $\phi^{n}\in H^{2}(\Omega )$*, there exists a solution* $\phi^{n+1}$ *satisfying the backward Euler scheme* (20). *Moreover,* $\phi^{n+1}$ *may be given by the minimizer in* $H^{2}(\Omega )$ *of the modified energy functional:*


$$E_{M}(\phi )+\frac{1}{2\Delta t}\int_{\Omega }(\phi -\phi^{n}{)}^{2}dx.$$ (21)

*Furthermore,*


$$E_{M}(\phi^{n+1})-E_{M}(\phi^{n})+\frac{1}{2\Delta t}\int_{\Omega }(\phi^{n+1}-\phi^{n}{)}^{2}dx\leq 0.$$

The proof is adapted from the similar result in Du and Wang [21].

## Results

### Numerical accuracy and energy stability

Before systematically investigating the morphological transitions, we first validate the numerical accuracy and energy stability of the proposed semi-implicit Fourier-spectral scheme.

**Temporal Convergence:** Since an exact analytical solution for this highly non-linear phase-field model is unavailable, we perform a Cauchy-type convergence test to determine the temporal accuracy. Specifically, we calculated the *L*^2^ error between numerical solutions obtained with two successive time steps, defined as $e_{\Delta t}=||\phi_{\Delta t}-\phi_{\frac{\Delta t}{2}}|{|}_{L^{2}}$. The corresponding convergence order is calculated by $\log_{2}\left(\frac{e_{\Delta t}}{e_{\frac{\Delta t}{2}}}\right)$. We choose a fixed, sufficiently fine spatial grid to isolate the temporal discretization error. As summarized in Table 1, the numerical scheme exhibits a strict asymptotic first-order accuracy in time (approaching 0.9995), which is completely consistent with the theoretical expectation of our semi-implicit temporal discretization (14). Furthermore, regarding the spatial accuracy, our method inherently achieves exponential (spectral) convergence for smooth solutions due to the properties of the Fourier-spectral approximation.

**Table 1:** Temporal convergence rate of *ϕ.*

Δt	*Error*	*Order*
2.00*e* − 7	8.241*e* − 5	–
1.00*e* − 7	4.132*e* − 5	0.9961
5.00*e* − 8	2.069*e* − 5	0.9981
2.50*e* − 8	1.035*e* − 5	0.9990
1.25*e* − 8	5.177*e* − 6	0.9995

**Fig 1:**
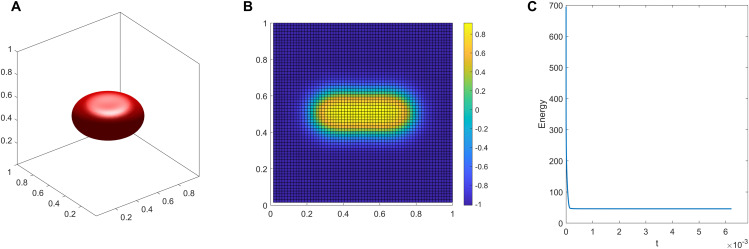
Discocyte shape. **A**:3D view. **B**:front view. **C**:energy evolution.

**Fig 2:**
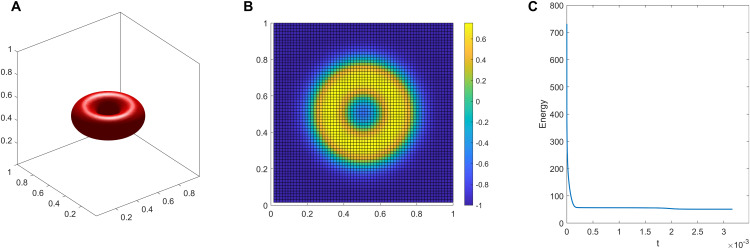
Torus shape. **A**:3D view. **B**:top view. **C**:energy evolution.

**Energy Stability:** To numerically verify the energy dissipation property of our scheme, we track the evolution of the total discrete energy during the simulations. As representative examples, Figs 1 and 2 display the energy evolution curves for Experiments (1) and (2). These curves intuitively demonstrate that the total energy decays monotonically over time and eventually plateaus, indicating that the system has successfully reached a stable equilibrium. It should be noted that all other simulated morphologies presented in this work are likewise final steady-state results; however, their corresponding energy curves are omitted here for the sake of brevity. This strict monotonic decay physically confirms the robust energy stability of our numerical framework, even when handling the severe stiffness induced by the ADE functional.

In this section, we present a series of numerical experiments to demonstrate the capabilities of the proposed model. All simulations are performed in a cubic domain Ω with periodic boundary conditions. The spatial variables are discretized using a Fourier-spectral method with a cubic grid of grid size *h*. The temporal evolution is solved using the semi-implicit scheme described in (14). This scheme improves stability and allows for larger time steps, making it more efficient than fully explicit methods, especially for long-time simulations. Unless otherwise specified, the model parameters are set as follows: $\Omega =[0,1{]}^{3}$, $h=\frac{1}{64}$, κ=1, $\bar{\kappa }=1.4$, *M*_1_ = 10^5^ and *M*_2_ = 10^4^. For the numerical experiments, we only considered *C* = 0 to showcase the influence of the ADE term only. The physical distance between leaflets is defined as $D=\frac{2}{3}\epsilon$, where ϵ is the interface width. Each simulation is run until the system reaches a steady state.

To quantitatively characterize the simulated vesicle morphologies and facilitate direct comparison with classical theoretical phase diagrams [22], we introduce two fundamental dimensionless parameters: the reduced volume *v* and the reduced area difference $\Delta a_{0}$. For a vesicle with a given volume α and surface area β, the equivalent radius of a sphere with the same surface area is defined as $R_{s}=\sqrt{\frac{\beta }{4\pi }}$. The reduced volume and reduced area difference are then given by:


$$v=\frac{\alpha }{\frac{4\pi }{3}R_{s}^{3}},\;\Delta a_{0}=\frac{\Delta A_{0}}{8\pi DR_{s}}.$$

In the following experiments, we will systematically track these two parameters alongside the morphological transitions.

### Formation of classic vesicle shapes

(1) Let ϵ=0.04, $\Delta t=1\times 10^{-6}$, *v* = 0.8, $\Delta a_{0}=1.2$. The initial condition of the phase-field variable is given as

$$u_{0}=\tanh\left(\frac{0.35-(\frac{(x-0.5{)}^{2}}{0.5}+\frac{(y-0.5{)}^{2}}{0.5}+\frac{(z-0.5{)}^{2}}{0.1})}{\sqrt{2}\epsilon }\right).$$ (22)

The final steady-state configuration is a discocyte shape illustrated in Fig 1. Fig 1A shows the 3D view of the shape, while Fig 1B presents its corresponding cross-section. The cross-section clearly shows that the phase-field variable transitions smoothly between −1 and 1, which validates the diffuse interface nature of our model. This behavior of the phase field is similar for all examples considered in this paper. Fig 1C, showing the time evolution of the energy, demonstrates that the system ultimately reaches a stable equilibrium state.

(2) Let *v* = 0.8, $\Delta a_{0}=1.4$, Other parameters follow the same configuration as (1). The final steady-state configuration is a torus. Its 3D view, top view and energy evolution are illustrated in Fig 2A, 2B and 2C, respectively.
(3) Next, we investigate the continuous morphological transitions driven by systematically varying the reduced area difference $\Delta a_{0}$. In this series of experiments, the initial state is an ellipsoid (23). We fix the reduced volume at *v* = 0.9 and maintain other core parameters constant (ϵ=0.02, $\Delta t=5\times 10^{-7}$).

$$u_{0}=\tanh\left(\frac{0.5-(\frac{(x-0.5{)}^{2}}{0.2^{2}}+\frac{(y-0.5{)}^{2}}{0.2^{2}}+\frac{(z-0.5{)}^{2}}{0.35^{2}})}{\sqrt{2}\epsilon }\right).$$ (23)

**Fig 3:**
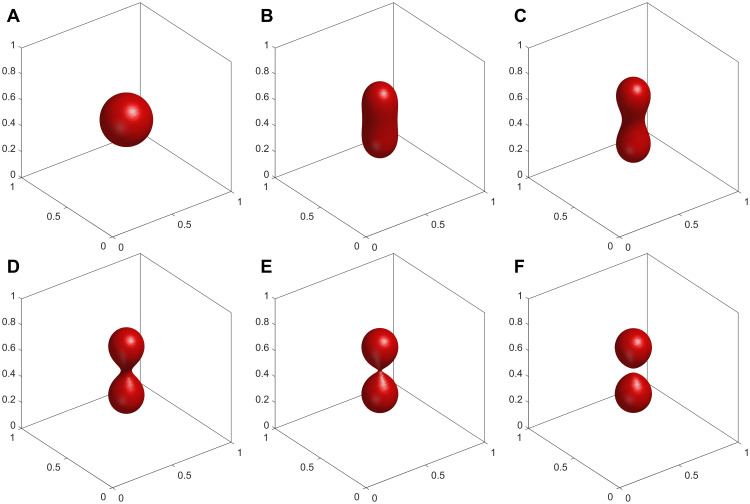
Morphological transition of a vesicle from a spherical shape to complete fission with increasing reduced area difference $\Delta a_{0}$ at a fixed reduced volume v=0.9. **A**:$\Delta a_{0}=1.1$. **B**:$\Delta a_{0}=1.4$. **C**:$\Delta a_{0}=1.5$. **D**:$\Delta a_{0}=1.6$. **E**:$\Delta a_{0}=1.7$. **F**:$\Delta a_{0}=1.8$.

As illustrated in Fig 3, our numerical results closely parallel the shape deformation trajectories observed in recent experiments, where an internal chemical trigger significantly increases $\Delta a_{0}$ to drive vesicle budding and eventual division [25]. The shape transformation begins from a nearly spherical shape at $\Delta a_{0}=1.1$ (Fig 3A). As $\Delta a_{0}$ incrementally increases, the vesicle elongates into a prolate shape at $\Delta a_{0}=1.4$ (Fig 3B) and further constricts at the equator to form dumbbell-like intermediate shapes (Figs 3C and 3D). At $\Delta a_{0}=1.7$, the vesicle reaches the budded limiting shape consisting of two connected spheres with a microscopic neck (Fig 3E), which aligns with the theoretical budding limit. Most notably, when $\Delta a_{0}$ is further increased to 1.8, our phase-field framework naturally captures the subsequent topological transition: the narrow neck completely ruptures, resulting in the successful fission of the membrane into two disconnected daughter vesicles (Fig 3F). This simulated trajectory robustly demonstrates that our model can seamlessly capture both the continuous shape deformations and the discontinuous topological division processes observed in biological and artificial systems [25].

### Emergence of complex, high-genus morphologies

**Fig 4:**
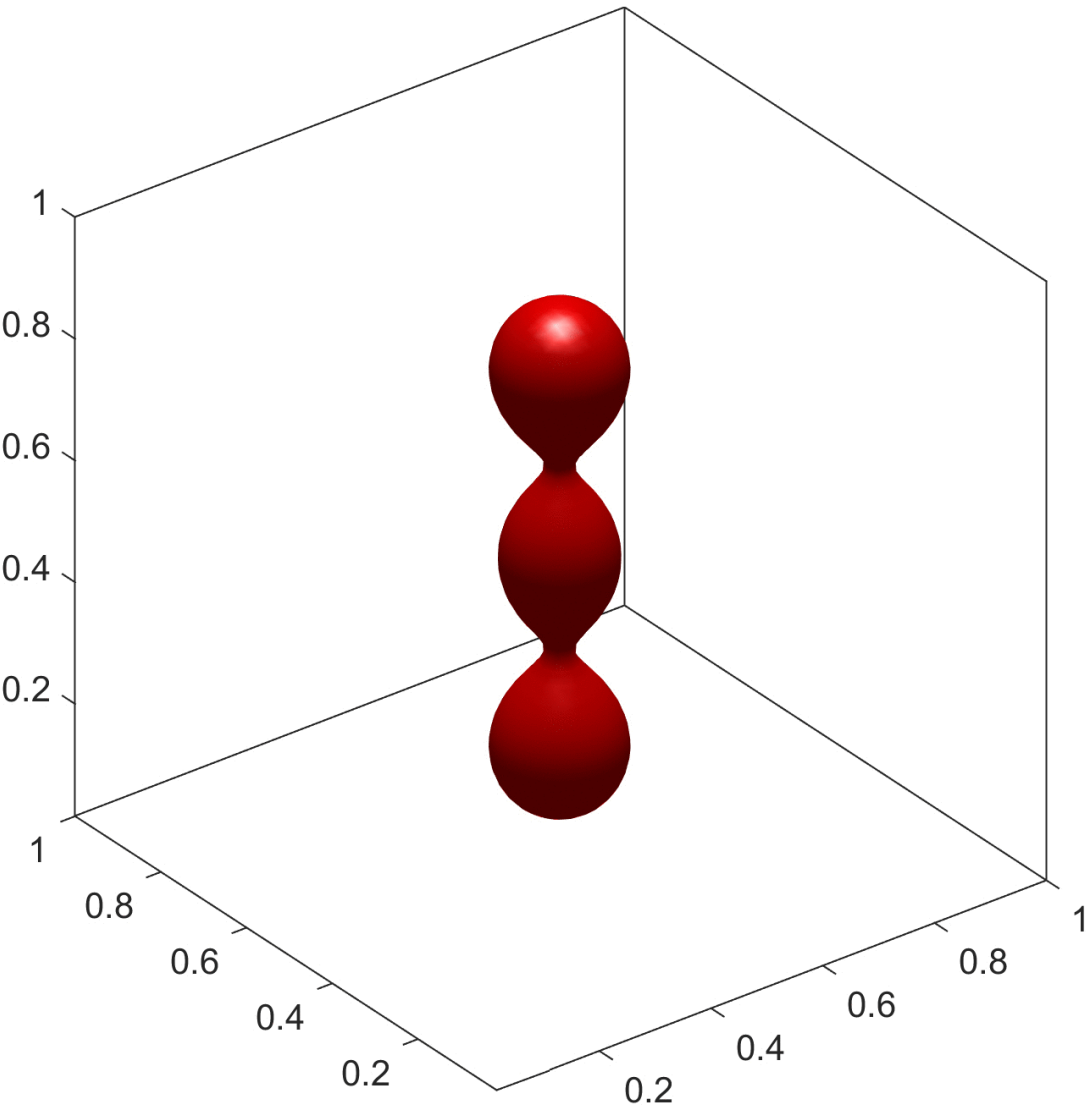
Chain shape.

**Fig 5:**
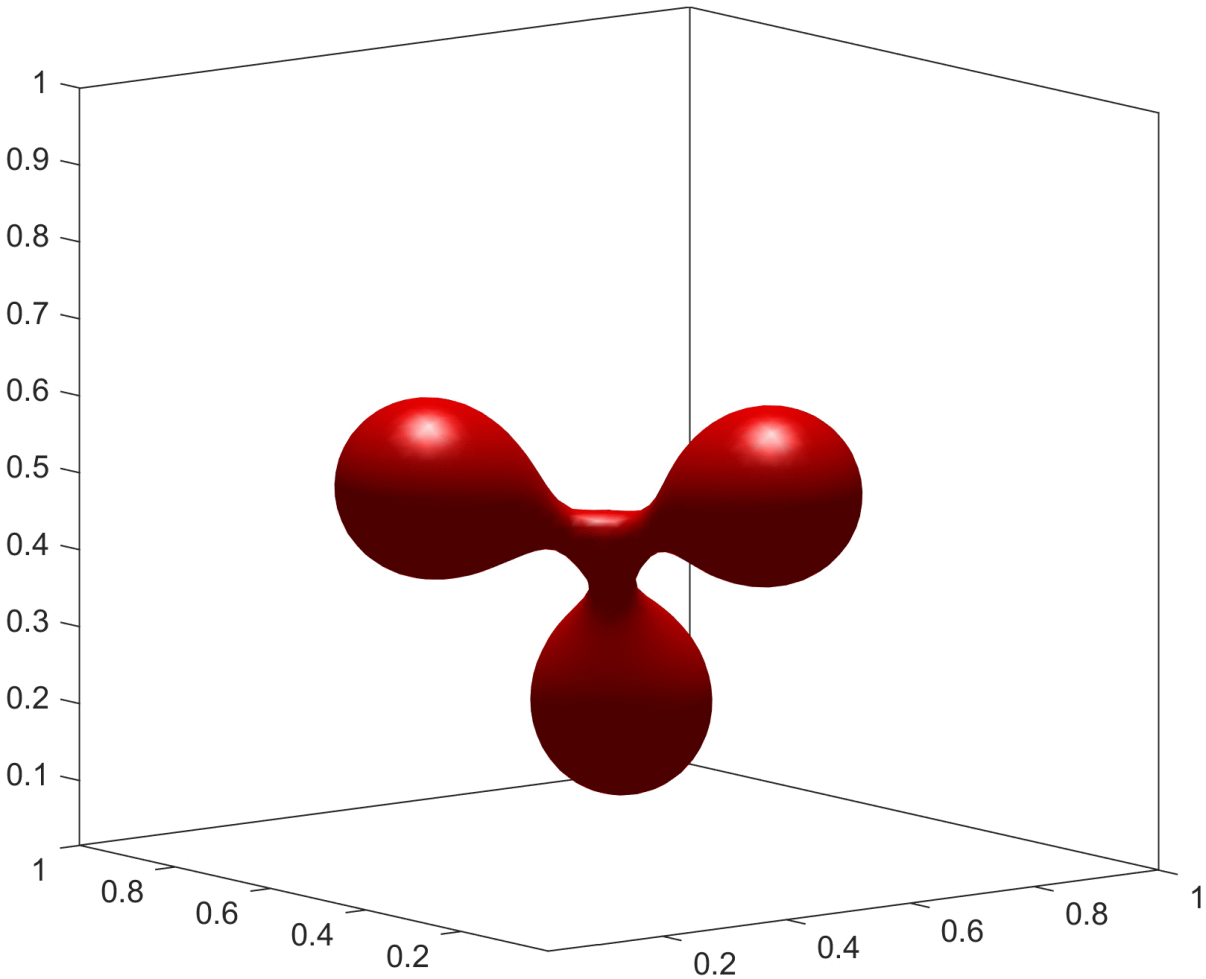
Three-armed shape.

**Fig 6:**
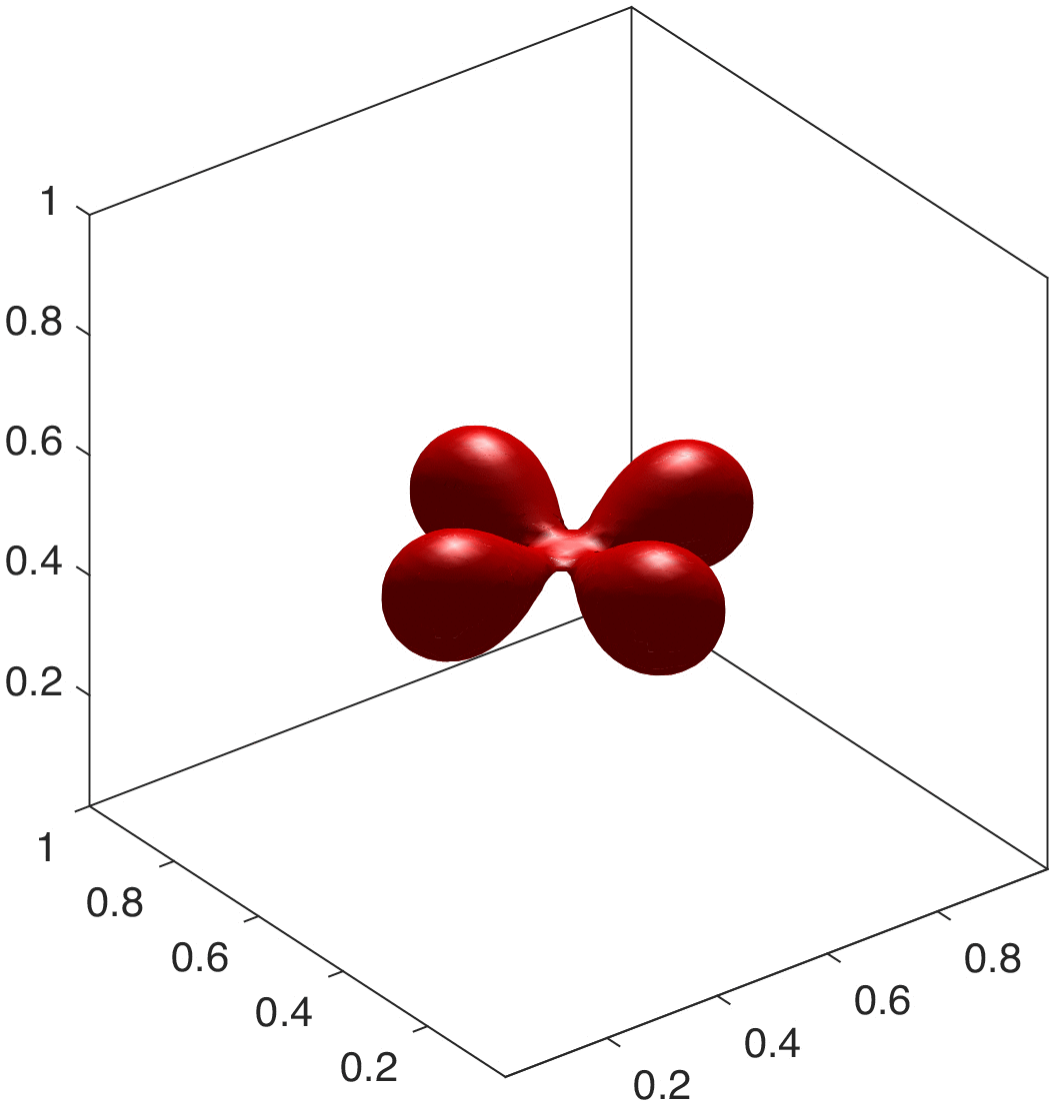
Four-armed shape.

**Fig 7:**
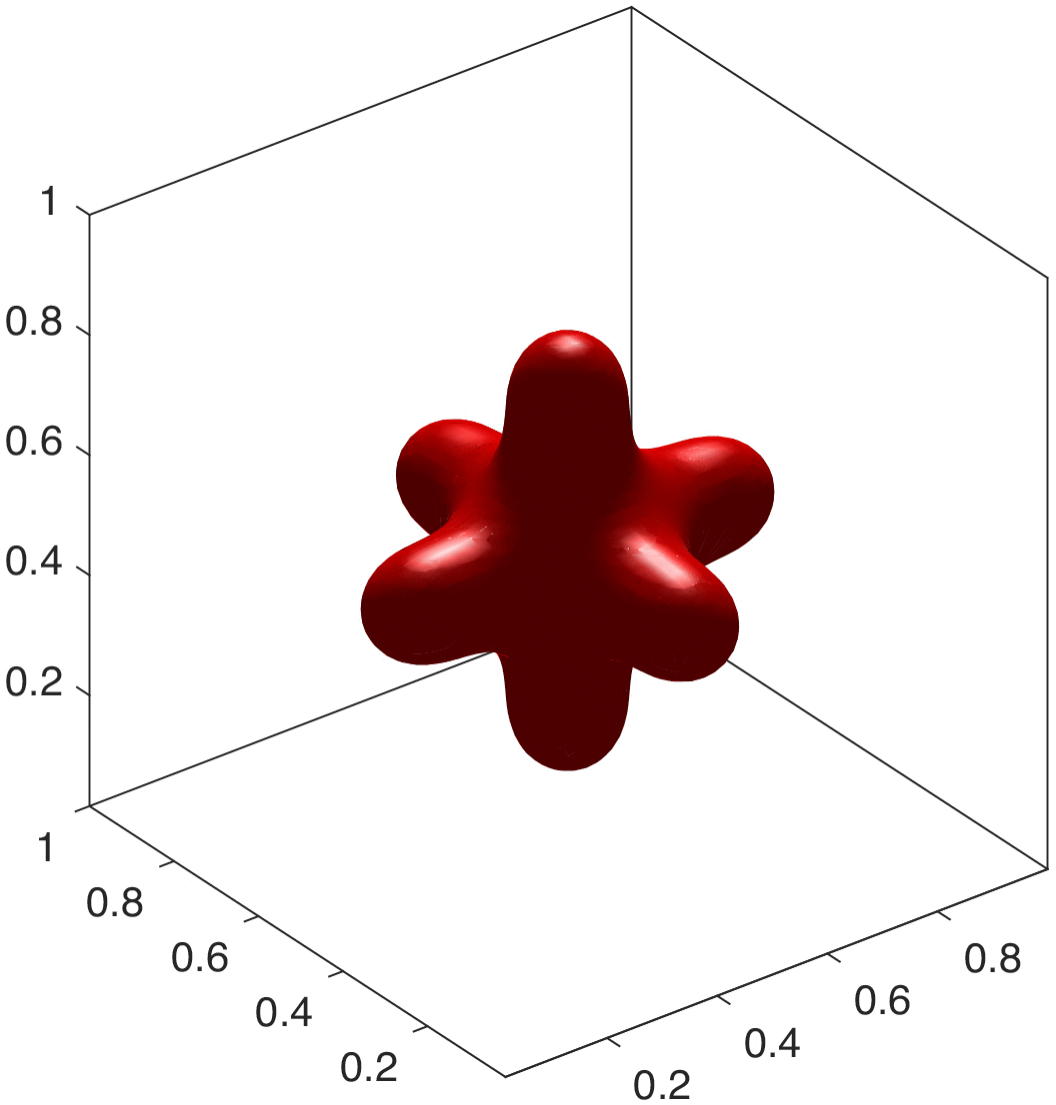
Six-armed shape.

To showcase the model’s versatility, we explored parameter regimes that give rise to more complex topologies. We found that these intricate, high-genus shapes are more readily formed with a smaller interface thickness (ϵ=0.02). A smaller ϵ provides a better approximation of the sharp-interface bending energy, particularly in regions of high curvature that are characteristic of such complex structures. By further adjusting the volume and area constraints, we can stabilize a rich spectrum of configurations. These include an elongated chain-like structure (Fig 4), which mimics vesicle fission. Furthermore, by adjusting the initial geometry and constraints, we can generate multi-armed, starfish-like configurations. Figs 5–7 show stable steady states with three, four, and six arms, respectively. The formation of these complex, high-genus structures highlights the model’s capability to capture sophisticated membrane behaviors beyond simple deformations. This makes it a powerful tool for exploring phenomena such as the formation of tubular networks in cellular organelles [26,27]. The specific parameters are detailed in experiments (4) to (8).

(4) Let ϵ=0.02, $\Delta t=5\times 10^{-7}$, *v* = 0.9, $\Delta a_{0}=1.4$. The initial condition is (23) and the final steady-state configuration is a chain shape illustrated in Fig 4.
(5) Let ϵ=0.02, $\Delta t=1\times 10^{-7}$, *v* = 0.8, $\Delta a_{0}=1.4$. The initial condition is

$$u_{0}=\tanh\left(\frac{0.5-(\frac{(x-0.5{)}^{2}}{0.35^{2}}+\frac{(y-0.5{)}^{2}}{0.35^{2}}+\frac{(z-0.5{)}^{2}}{0.35^{2}})}{\sqrt{2}\epsilon }\right).$$ (24)

The final steady-state configuration is a three-armed shape. The result is illustrated in Fig 5

(6) Let ϵ=0.02, $\Delta t=2\times 10^{-7}$, *v* = 0.8, $\Delta a_{0}=1.6$. The initial condition is

$$u_{0}=\tanh\left(\frac{0.5-(\frac{(x-0.5{)}^{2}}{0.35^{2}}+\frac{(y-0.5{)}^{2}}{0.35^{2}}+\frac{(z-0.5{)}^{2}}{0.15^{2}})}{\sqrt{2}\epsilon }\right).$$ (25)

The final steady-state configuration is a four-armed shape in Fig 6.

(7) Let ϵ=0.02, $\Delta t=1\times 10^{-7}$, *v* = 0.6, $\Delta a_{0}=1$. The initial condition is

$$u_{0}=\tanh\left(\frac{0.6-(\frac{(x-0.5{)}^{2}}{0.35^{2}}+\frac{(y-0.5{)}^{2}}{0.35^{2}}+\frac{(z-0.5{)}^{2}}{0.35^{2}})}{\sqrt{2}\epsilon }\right).$$ (26)

The final steady-state configuration is a six-armed shape illustrated in Fig 7.

We also explore the formation of nested shapes [24] within vesicle membranes. By adjusting initial condition, vesicles can form intricate configurations such as multi-chambered structures or internal spherical shapes. As shown in the following example:

(8) Let $\Omega =[0,2{]}^{3}$, $h=\frac{1}{50}$, ϵ=0.03, $\bar{\kappa }=4$, *M*_1_ = *M*_2_ = 10^4^, $\Delta t=5\times 10^{-7}$, *v* = 0.6, $\Delta a_{0}=1.1$. The initial condition is

$$u_{0}=\tanh\left(\frac{1-(\frac{(x-1{)}^{2}}{0.16}+\frac{(y-1{)}^{2}}{0.16}+\frac{(z-1{)}^{2}}{0.16})}{\sqrt{2}\epsilon }\right).$$ (27)

**Fig 8:**
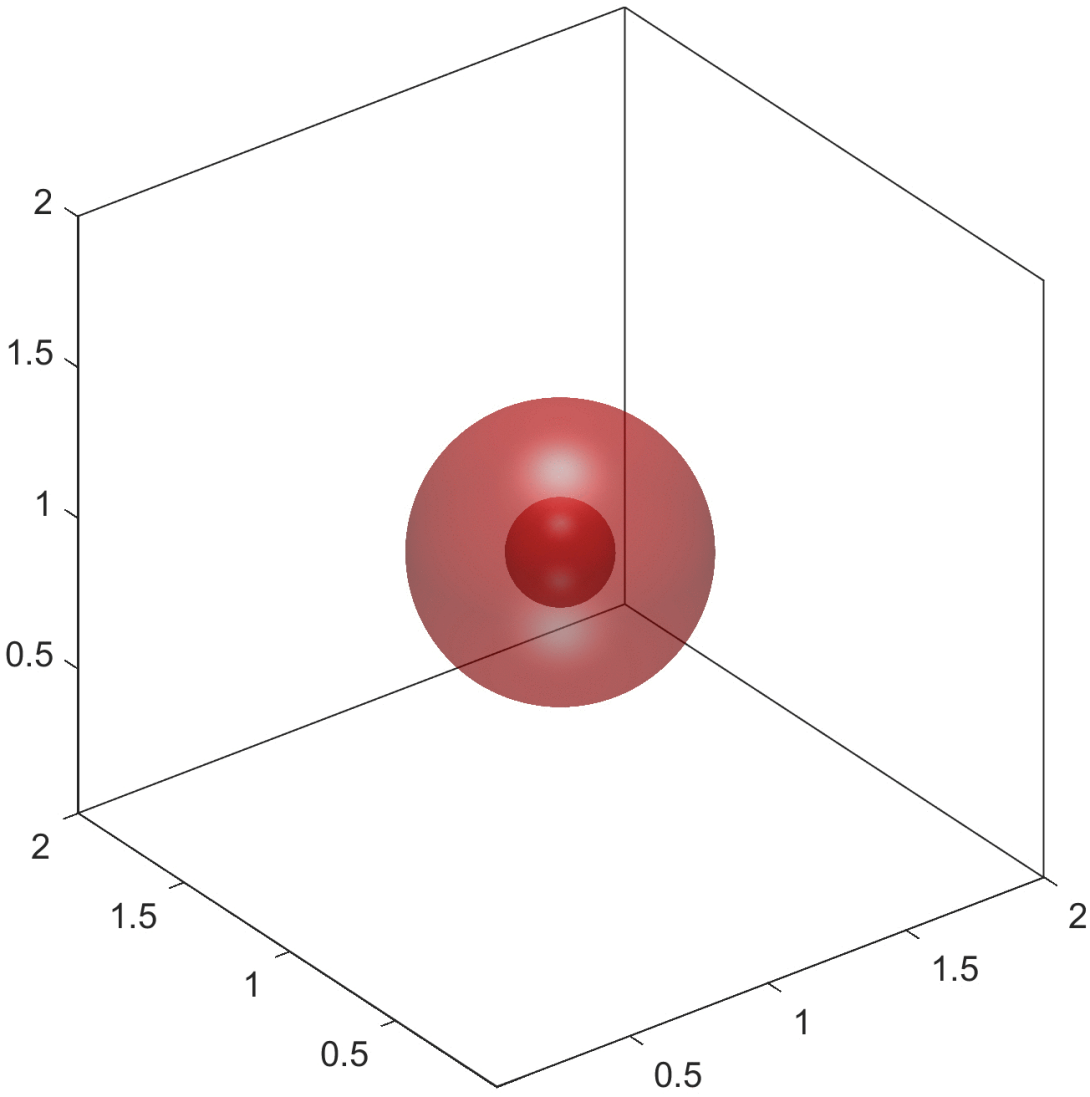
Nested shape.

The final steady-state configuration emerges where two spheres are embedded within one another, forming a nested vesicle (Fig 8). This complex topology showcases how the interplay between membrane constraints can lead to novel geometries, which may be relevant for modeling vesicle fission or cell division processes.

### Quantitative validation and phase diagram

**Fig 9:**
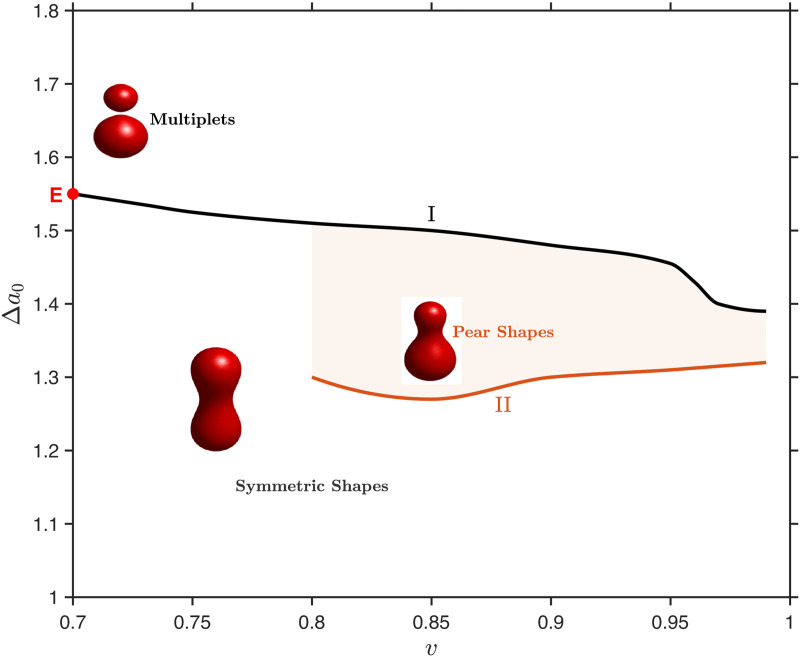
Phase diagram in the $\left(v,\Delta a_{0}\right)$ plane.

By tracking the dimensionless parameters *v* and $\Delta a_{0}$ defined above, we mapped our steady-state 3D simulations onto a global phase diagram. As illustrated in Fig 9, our phase-field model successfully reproduces the characteristic morphological regions and symmetry-breaking transitions predicted by classical theories [22].

Notably, through delicate parameter tuning, our robust framework successfully resolves the asymmetric pear shape region, which is bounded by curve I (the transition to vesiculated multiplets) and curve II (the symmetry-breaking boundary from symmetric shapes). Specifically, curve I terminates at point E, which represents the theoretical limiting shape consisting of two connected spheres of equal radii, perfectly aligning with the definition in Miao et al. [22]. It is worth mentioning that capturing this specific asymmetric shape requires navigating a complex energy landscape with multiple local minima. Unlike the symmetric morphologies that readily evolved from a standard initial ellipsoid, initiating the simulation with an ellipsoid in this regime often leads the system to become trapped in a metastable symmetric state. To overcome this, we utilized a deliberately asymmetric initial configuration constructed by the union of two unequal spheres (a larger sphere at the bottom and a smaller sphere at the top). Let *d*_1_ and *d*_2_ represent the distances from any point (*x*,*y*,*z*) to the centres of the bottom and top spheres, respectively:


$$d_{1}=\sqrt{(x-0.5{)}^{2}+(y-0.5{)}^{2}+(z-0.4{)}^{2}},\;d_{2}=\sqrt{(x-0.5{)}^{2}+(y-0.5{)}^{2}+(z-0.7{)}^{2}}.$$

The initial phase-field profile is then mathematically expressed as


$$u_{0}=\tanh\left(\frac{\text{max}(0.22-d_{1},0.4-d_{2})}{\sqrt{2}\epsilon }\right).$$ (28)

It is crucial to emphasize that the stabilization of this asymmetric pear shape is fundamentally driven by the area-difference elasticity. Recent studies utilizing phase-field models governed solely by bending energy with strict volume and surface area constraints—but lacking the ADE term —have shown that even highly asymmetric initial configurations (such as the collision of multiple non-concentric vesicles) invariably relax into axisymmetric steady-state shapes [28]. Therefore, the inclusion of the ADE functional is strictly required to break this symmetry and sustain the pear-shaped morphology. Furthermore, a parameter continuation strategy was employed: the stable steady-state solution obtained from a neighbouring parameter set was utilized as the initial condition for the subsequent simulation. Within the pear-shaped region, as $\Delta a_{0}$ increases for a fixed reduced volume *v*, the vesicle smoothly breaks its up-down reflectional symmetry, crossing curve II to form an asymmetric pear, and eventually undergoes hemifusion into multiplets by crossing curve I.

It is important to highlight the fundamental physical distinctions between our phase-field results and classical sharp-interface diagrams. In our framework, minor discrepancies in the exact location of the boundaries—such as the absence of stable pear shape for *v* < 0.8, where symmetric shapes transition directly into multiplets—can be directly attributed to the diffuse-interface nature of the phase-field method. The finite interfacial thickness ϵ introduces an intrinsic energetic penalty at regions of extreme local curvature (such as the narrow neck of a highly deflated pear). This effectively regularizes the mathematical singularity and offers a physically realistic representation of lipid bilayers with non-zero thickness.

## Discussion

### Physical interpretation of complex topologies and nested configurations

The emergence of nested configurations in our numerical simulations raises important physical and topological considerations. The standard ADE theory is primarily formulated for a single, topologically closed bilayer. However, as demonstrated experimentally by Salva et al. [24], fluid vesicles subjected to volume reduction undergo a continuous shape evolution from a stomatocyte to a nested vesicle. This transformation occurs when the invaginated parts of a stomatocyte approach each other, come into close contact, and eventually fuse. Therefore, the resulting nested configuration consists of surfaces that originated from a single continuous parent membrane, sharing the total membrane surface area and the enclosed internal volume.

The phase-field formulation is particularly advantageous in this context, as it inherently accommodates spontaneous topological changes, such as membrane fusion, which are mathematically challenging for sharp-interface parameterizations. When the vesicle undergoes hemifusion and splits into topologically disconnected surfaces, the global integration of the area difference ΔA over the entire computational domain Ω (as defined in Equation 10) naturally transitions to compute the sum of the area differences of the distinct surfaces. From a physical perspective, applying a single global ADE constraint to these disconnected surfaces is valid because it enforces the conservation of the intrinsic bending elastic moduli and the material properties of the parent vesicle across the newly formed multi-compartment system. This global energetic penalty successfully guides the vesicle through the complex topological transition and stabilizes the final nested morphology observed in experiments.

## Conclusion

In this paper, we developed and numerically implemented a phase-field model for vesicle membranes that incorporates area-difference elasticity while enforcing constraints on volume and surface area. Our model, solved by an efficient spectral method, successfully predicts a wide range of equilibrium morphologies. The numerical results demonstrate a clear pathway of shape transformations from simple spherical and discocyte shapes to complex structures like symmetric dumbbells, asymmetric pear shapes, complete membrane fission, multi-armed and nested configurations, primarily governed by the competition between bending energy and the ADE constraint. Future work will focus on further analysis of the capabilities of the proposed model by adopting biologically relevant dimensions of vesicles. As mentioned in [24], the sharp interface models fail to capture some experimentally observed steady-state shapes of the vesicles when the vesicle radius is below a threshold value around four times the membrane thickness. One such shape is the nested vesicle, which our formulation was able to capture effectively. We anticipate this to be due to the diffuse interface thickness having a biological relevance, which will be further explored. In addition, the proposed model will be further developed to include the phase field formulation of membrane cytoskeleton properties, essential for capturing the steady-state shapes of red blood cells [29].
